# Supplementary material for: Microbial synthesis of Prussian blue for potentiating checkpoint blockade immunotherapy
Source: Nat Commun. 2023 May 23;14:2943. doi: 10.1038/s41467-023-38796-9 (PMC10205718; doi:10.1038/s41467-023-38796-9)
Supplement: Supplementary file 1 — Supplementary Information [file 41467_2023_38796_MOESM1_ESM.pdf]

Supplementary Information

Microbial synthesis of Prussian blue for potentiating checkpoint blockade immunotherapy

Wang et al.

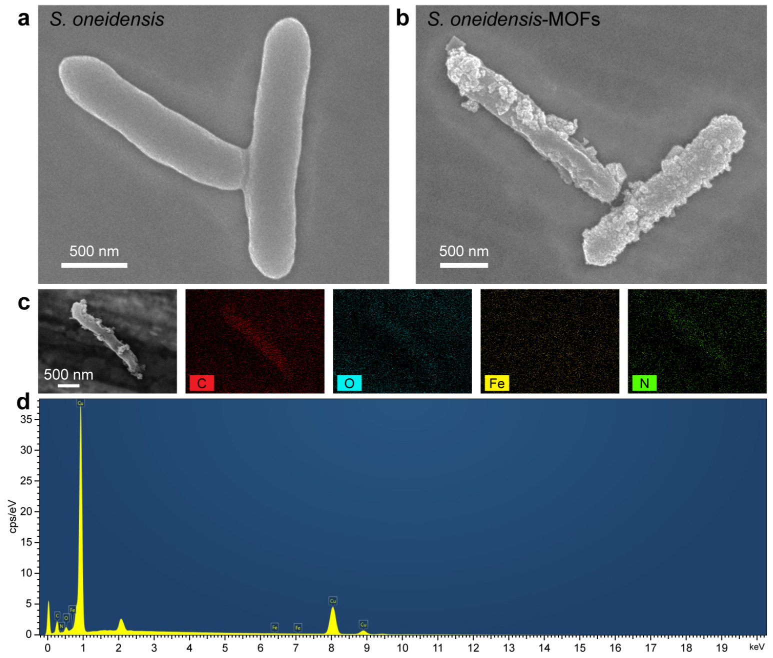

Supplementary Fig. 1. Characterization of *S. oneidensis* MR-1 and *S. oneidensis*-Prussian blue MOFs (*S. oneidensis*-MOFs). a-b, SEM images of *S. oneidensis* MR-1 and *S. oneidensis*-MOFs, respectively. **c,** SEM-EDX element mapping of C, O, Fe, and N for *S. oneidensis*-MOFs. **d,** Corresponding EDX spectrum of *S. oneidensis*-MOFs. The signal of copper should be assigned to the supported copper grid. The experiments were repeated three times with similar results.

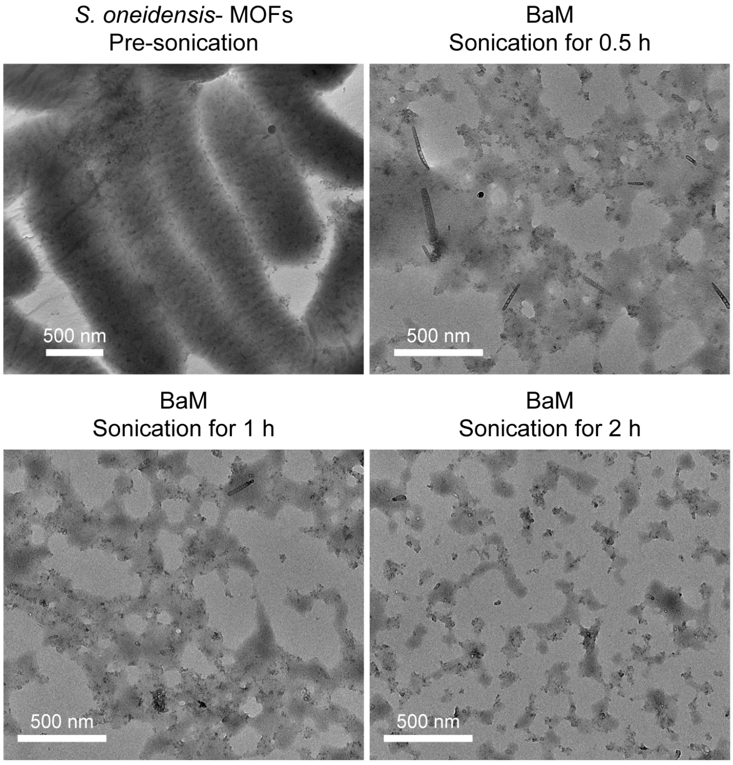

Supplementary Fig. 2. Characterization of the resulting BaM via sonication. TEM images showing time-dependent size and morphology changes with the prolonged sonication treatment of *S. oneidensis*-MOFs. The experiments were repeated three times with similar results.

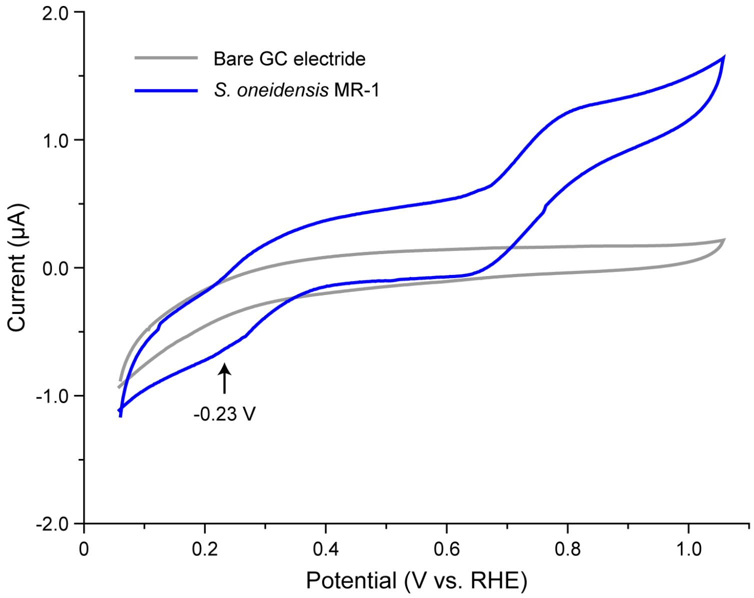

Supplementary Fig. 3. Characterization of *S. oneidensis* MR-1. Cyclic voltammograms of bare glassy carbon (GC) electrode and washed *S. oneidensis* MR-1 cells coated on a GC electrode in the presence of lactate in a buffer. The experiments were repeated three times with similar results.

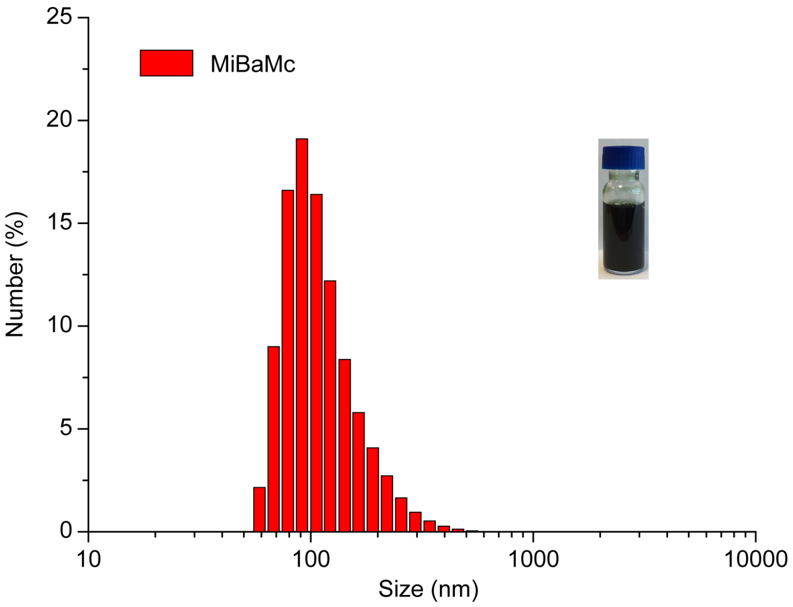

Supplementary Fig. 4. Characterization of MiBaMc. Hydrodynamic size distributions of the mitochondria-targeting MiBaMc were measured using dynamic light scattering. Insert shows the photograph of MiBaMc dispersed into saline. The experiments were repeated three times with similar results.

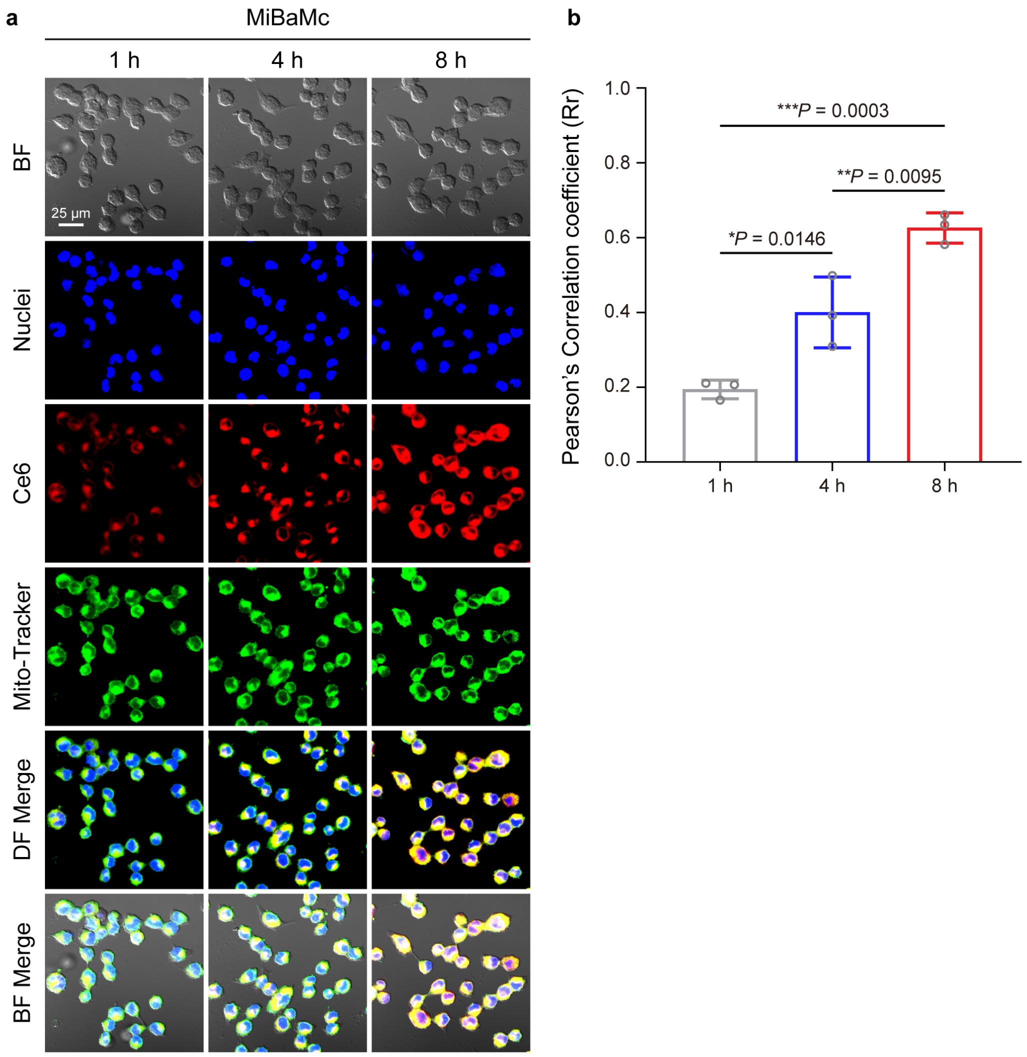

Supplementary Fig. 5. Time-dependent intracellular distribution of MiBaMc. a, Confocal images showing the intracellular distribution of MiBaMc nanoagents within 4T1 cancer cells with different incubation times. BF, bright field; DF, dark field. Mito-Tracker-stained mitochondria are shown in green, nuclei are stained with Hoechst 33342, and red fluorescence is from Ce6 of MiBaMc nanoagents. Scale bar, 25 µm. **b,** Statistic Pearson's correlation coefficient (Rr) from (**a**). Data are presented as mean values ± SD (n = 3 independent experiments). Statistical analysis was conducted using one-way ANOVA with Tukey's tests. $^{*}P < 0.05$, $^{**}P < 0.01$, $^{***}P < 0.001$.

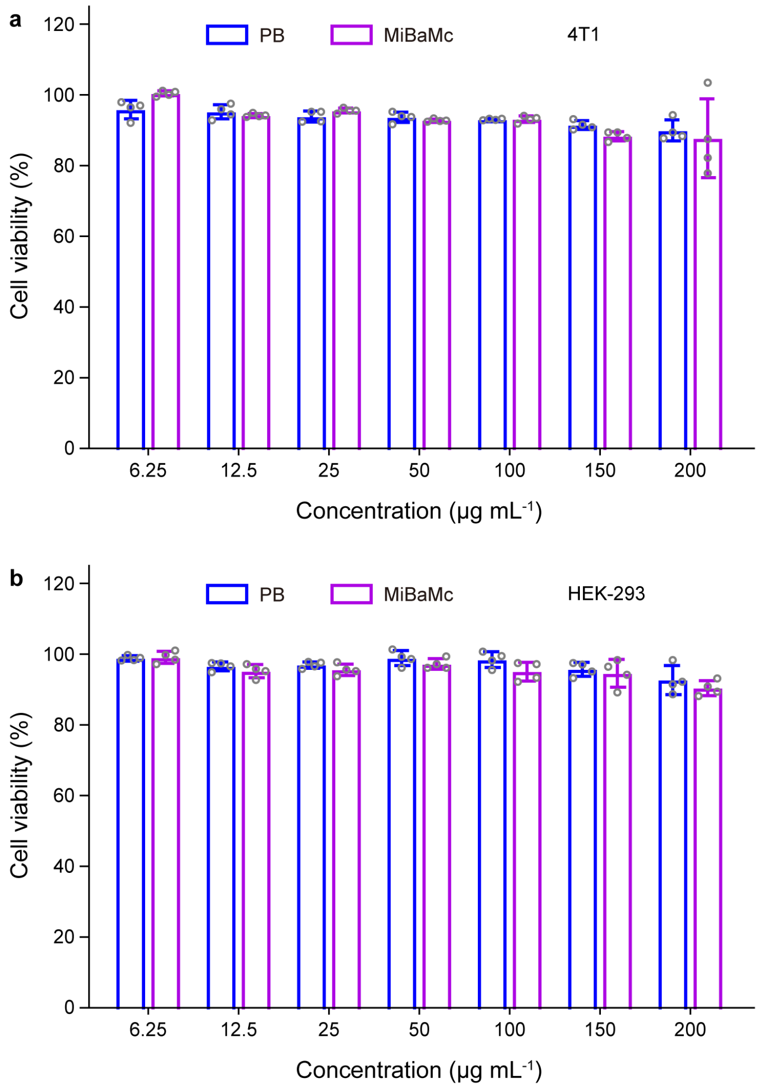

Supplementary Fig. 6. Biocompatibility of Prussian blue MOFs and MiBaMc (dark conditions) measured by MTT assay. a 4T1 cancer cells and **b** HEK-293 normal cells seeded into 96-wells plate at a concentration of 1 × 10^4 per well overnight. Prussian blue MOFs and MiBaMc nanoparticles at various concentrations were incubated with 4T1 and HEK-293 cells for 24 h under dark conditions before performing a standard methylthiazolyldiphenyl-tetrazolium bromide (MTT) assay. Data are presented as mean values ± SD (n = 4 independent experiments).

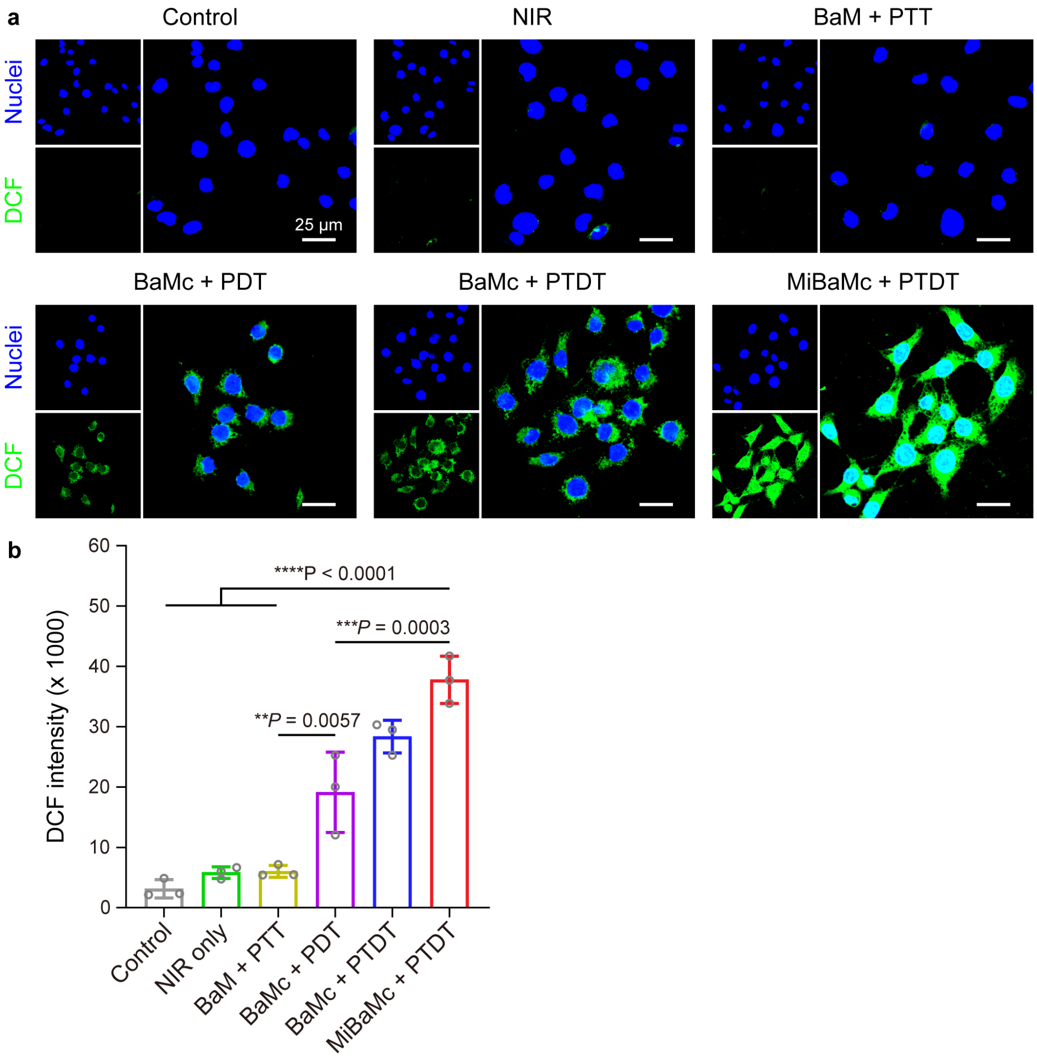

Supplementary Fig. 7. Intracellular ROS staining of 4T1 cells after different treatments. a, Confocal images of 4T1 cancer cells treated with PBS, NIR irradiation, BaM + PTT, BaMc + PDT, BaMc + PTDT, and MiBaMc + PTDT at a Ce6 concentration of 4 μg mL^{-1}. ROS levels were measured with the fluorescent probe DCFH-DA. Nuclei are stained with Hoechst 33342. Scale bar, 25 μm. **b,** Quantitative fluorescence intensity of DCF. Data are presented as mean values ± SD (n = 3 independent experiments). Statistical analysis was conducted using one-way ANOVA with Tukey's tests. $^{**}P < 0.01$, $^{***}P < 0.001$, $^{****}P < 0.0001$.

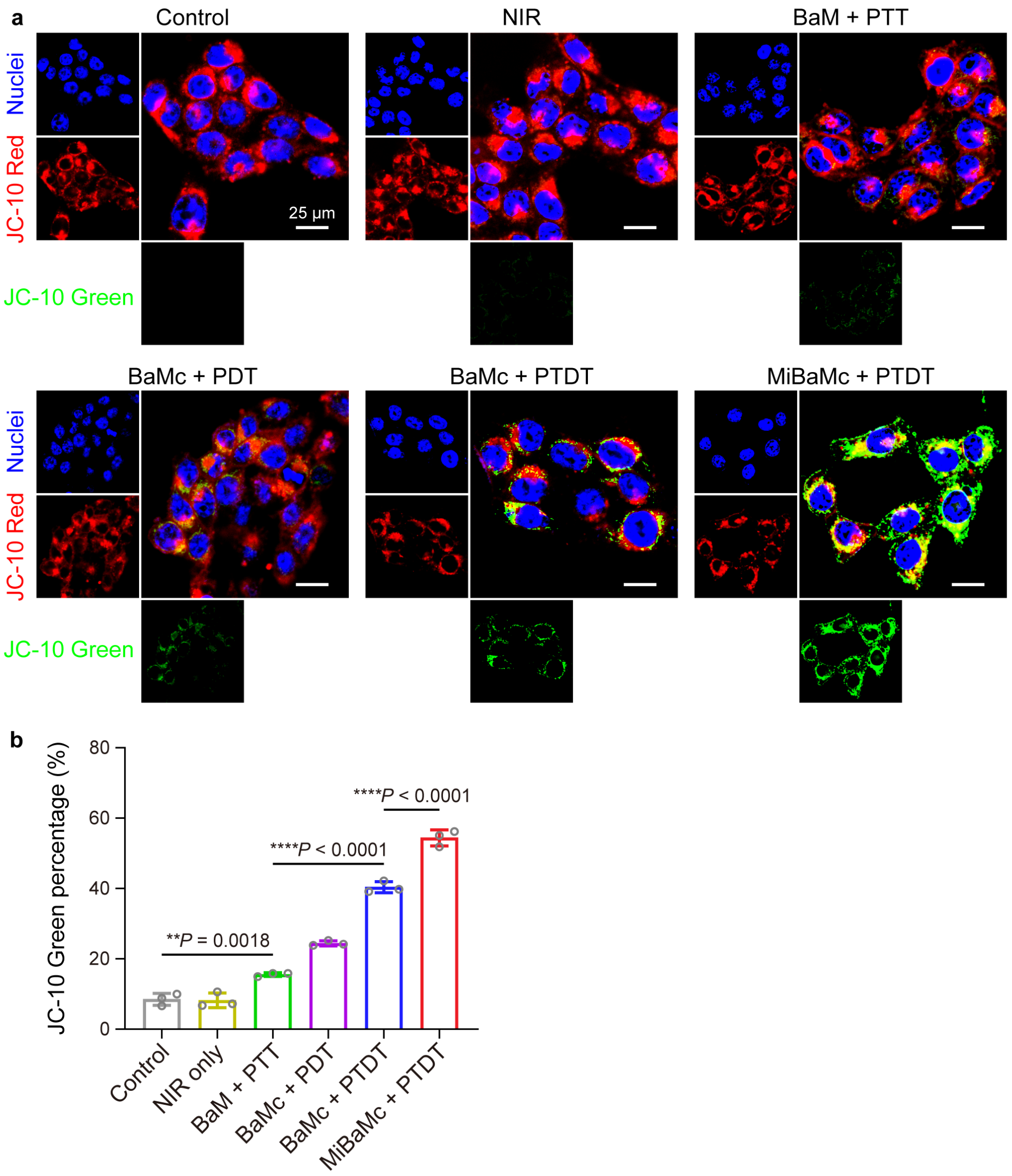

Supplementary Fig. 8. Mitochondrial membrane potential study of 4T1 cancer cells using JC-10 staining. a, Confocal images of 4T1 cancer cells treated with PBS, NIR irradiation, BaM + PTT, BaMc + PDT, BaMc + PTDT, and MiBaMc + PTDT at a Ce6 concentration of 4 μg mL^{-1}. The J-aggregates (normal cells) and monomeric forms (apoptotic and necrotic cells) after JC-10 staining were displayed in red and green, respectively. Scale bar, 25 μm. Nuclei are stained with Hoechst 33342. **b,** Quantitative percentage of the JC-10 green fluorescence. Data are presented as mean values ± SD (n = 3 independent experiments). Statistical analysis was conducted using one-way ANOVA with Tukey's tests. $^{**}P < 0.01$, $^{****}P < 0.0001$.

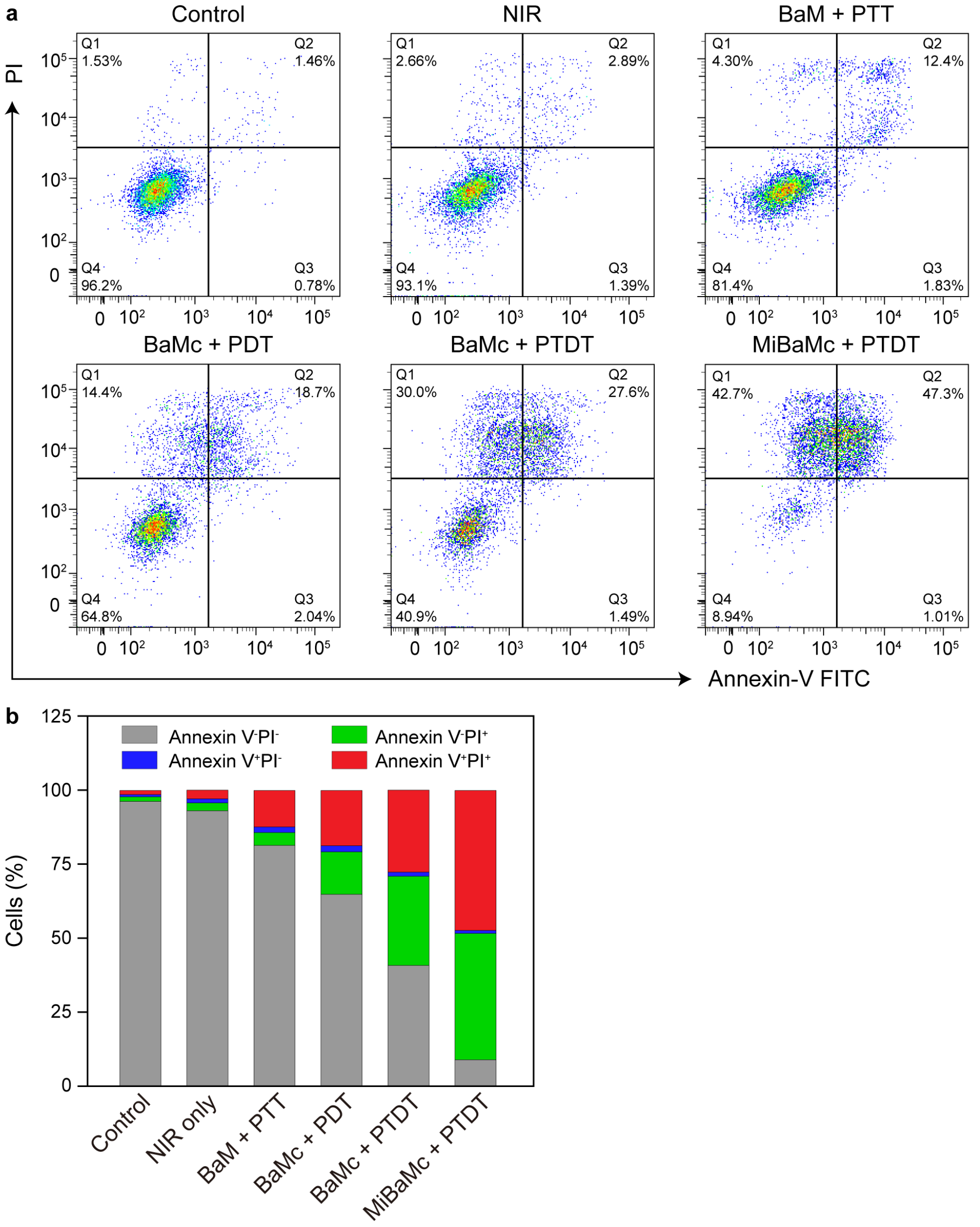

Supplementary Fig. 9. Apoptosis assay 24 h after different treatments. a, 4T1 cancer cells treated with PBS, NIR irradiation, BaM + PTT, BaMc + PDT, BaMc + PTDT, and MiBaMc + PTDT at a Ce6 concentration of 4 μg mL^{-1}. Cancer cells from different groups were co-stained with Annexin-V FITC and propidium iodide (PI) before performing flow cytometry. **b,** Different cell populations obtained from (**a**) apoptosis assay. The experiments were repeated three times with similar results.

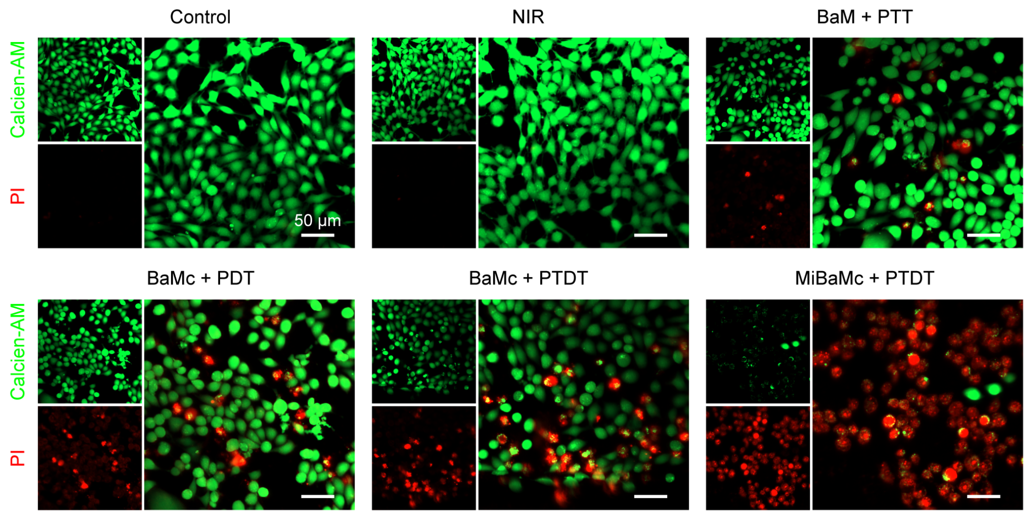

Supplementary Fig. 10. Dead/Live staining using confocal laser scanning microscope. Confocal images of 4T1 cancer cells treated with PBS, NIR irradiation, BaM + PTT, BaMc + PDT, BaMc + PTDT, and MiBaMc + PTDT at a Ce6 concentration of 4 μg mL^{-1}. The live cells and dead cells after calcein-AM/PI co-staining were displayed in green and red color, respectively. Scale bar, 50 μm. The experiments were repeated three times with similar results.

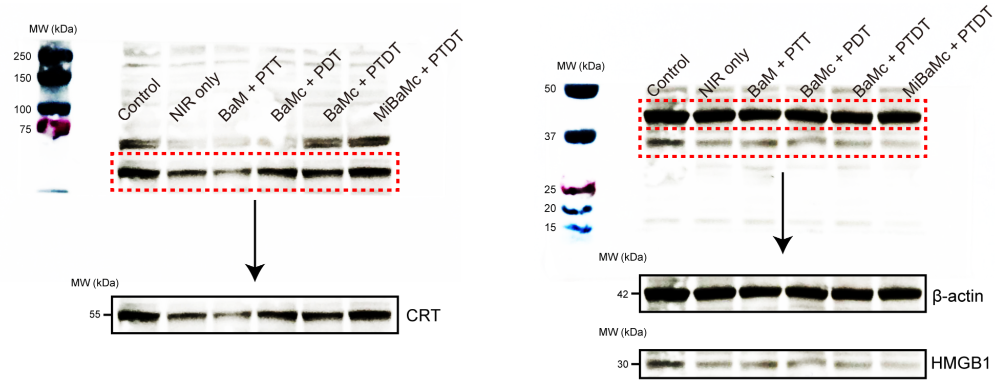

Supplementary Fig. 11. Western blots of CRT, HMGB1, and β-actin. Uncropped scans of all blots and gels of CRT, HMGB1, and β-actin expression in 4T1 cells treated under different conditions. Supplementary Fig. 11 is a supplementary note for Fig. 3g. The experiments were repeated three times with similar results.

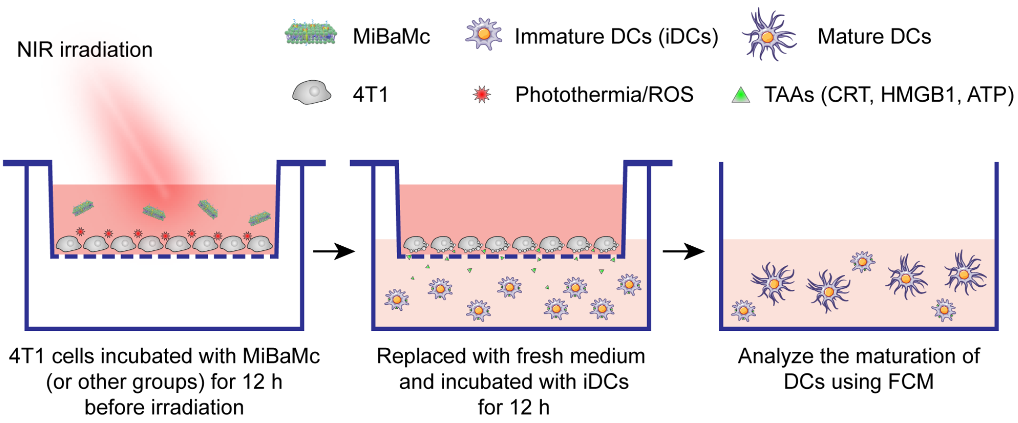

Supplementary Fig. 12. In vitro maturation of DCs triggered by MiBaMc. Schematic illustration of ICD-induced maturation of DCs in vitro.

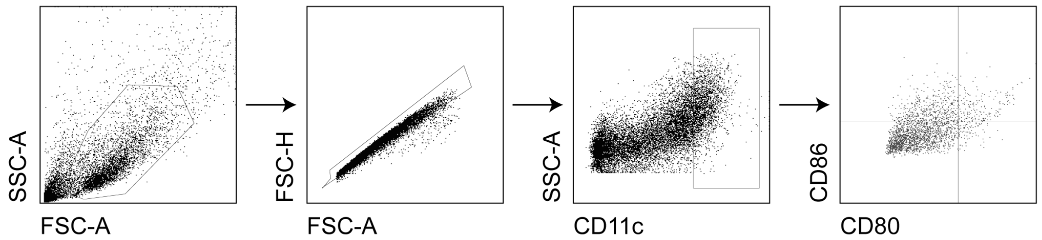

Supplementary Fig. 13. Gating strategies used for analyzing DCs maturation in vitro. Gating strategies to analyze matured DCs ($CD80^+$ and $CD86^+$) derived from bone marrow.

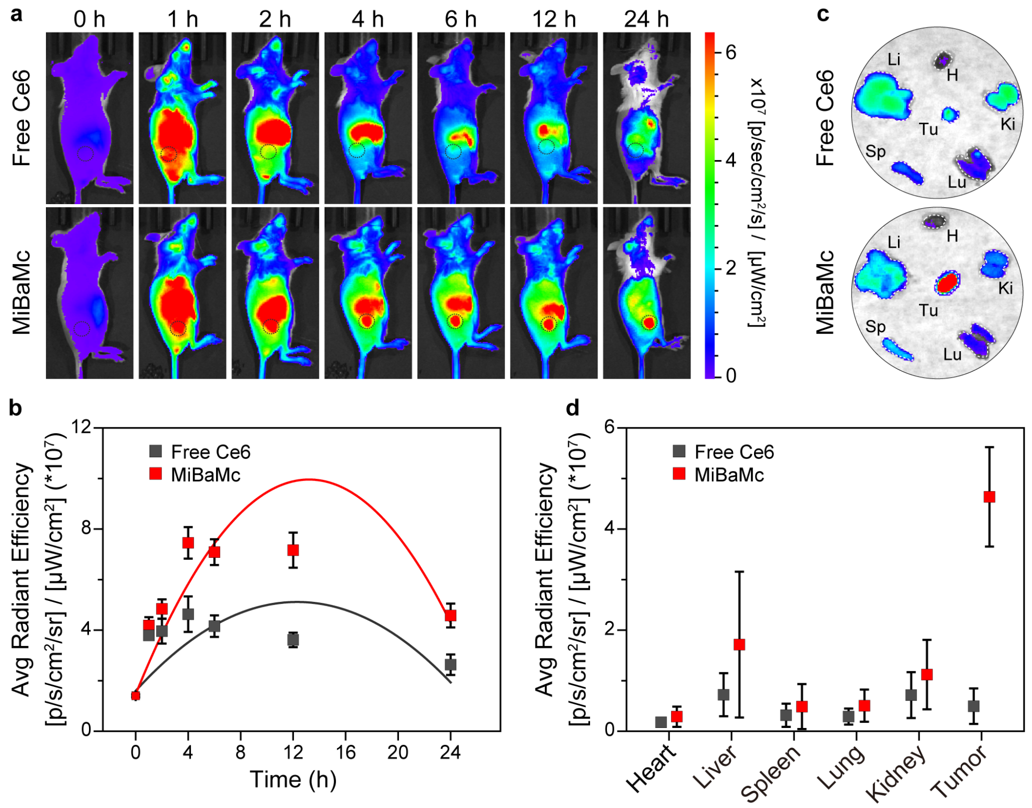

Supplementary Fig. 14. In vivo fluorescence imaging. a, 4T1 tumor-bearing mice intravenously injected with free Ce6 and MiBaMc at a Ce6 concentration of 4 mg kg^{-1}. Whole-body fluorescence images were captured before and after sample administration at pre-determined time points. **b,** Quantification of fluorescence signals in (**a**). **c,** Ex vivo fluorescence imaging of the main organs (heart, liver, spleen, lung, and kidney) and tumor tissues 24 h post-administration. **d,** Quantification of fluorescence signals in (**c**). (n = 3 mice).

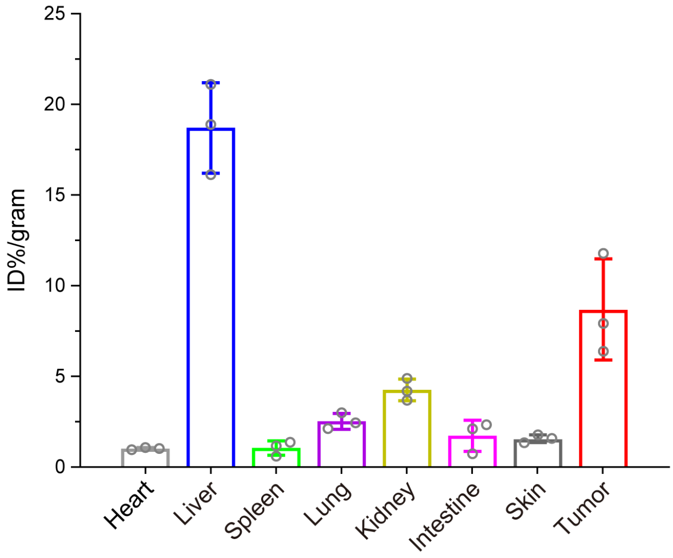

Supplementary Fig. 15. In vivo biodistribution of MiBaMc. Biodistribution of MiBaMc in the main organs 24 h post-administration (20 mg kg^{-1} based on MiBaMc) was determined using ICP-OES analysis. Data are presented as mean values ± SD (n = 3 mice).

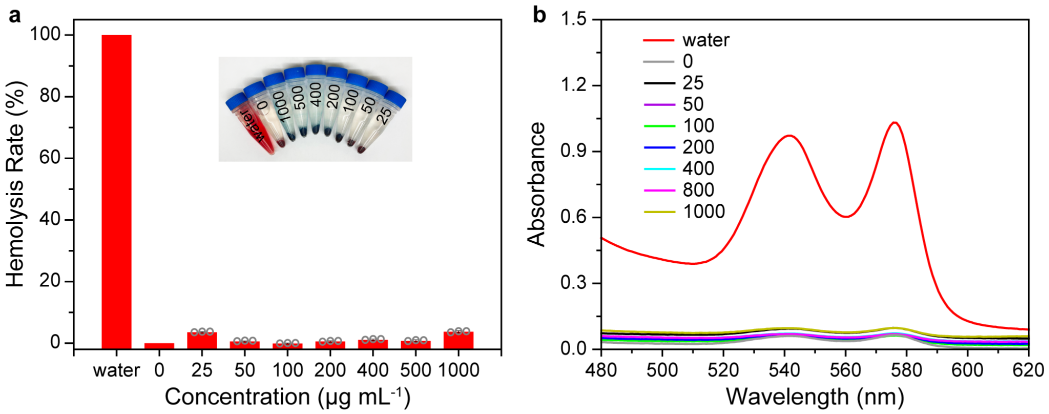

Supplementary Fig. 16. Hemolysis of red blood cells (RBC) after incubating with MiBaMc at different concentrations. a, Corresponding hemolysis rate after incubation with different concentrations of MiBaMc. **b,** UV-vis spectra of the corresponding supernatants. Inset in (**a**) is the photo of RBC-MiBaMc mixtures with different concentrations after centrifugation. Data are presented as mean values ± SD (n = 3 independent experiments).

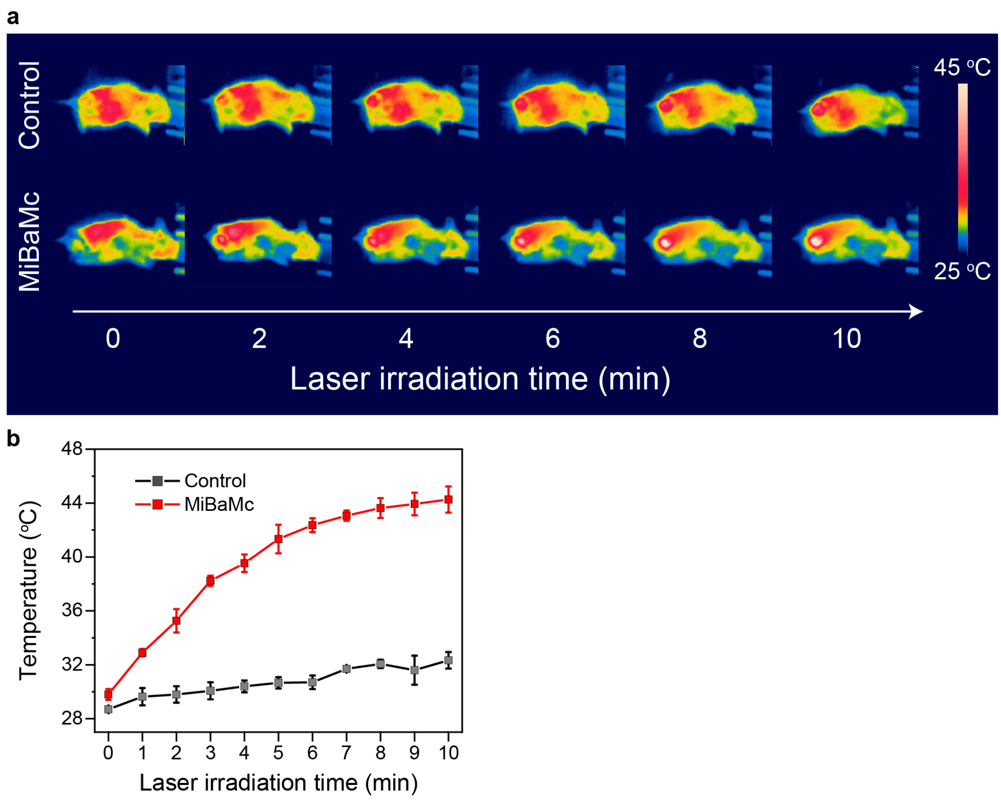

Supplementary Fig. 17. In vivo photothermal effect of MiBaMc. a, Thermal images of 4T1 tumor-bearing mice during 808 nm laser irradiation (1 W cm^{-2}) at 12 h post-administration of MiBaMc (200 μL per mouse, [Ce6] = 0.4 mg mL^{-1} [BaM] = 1.8 mg mL^{-1}). **b,** Maximum surface tumor temperature of 4T1 tumor-bearing mice during laser irradiation in (**a**). Data are presented as mean values ± SD (n = 3 mice).

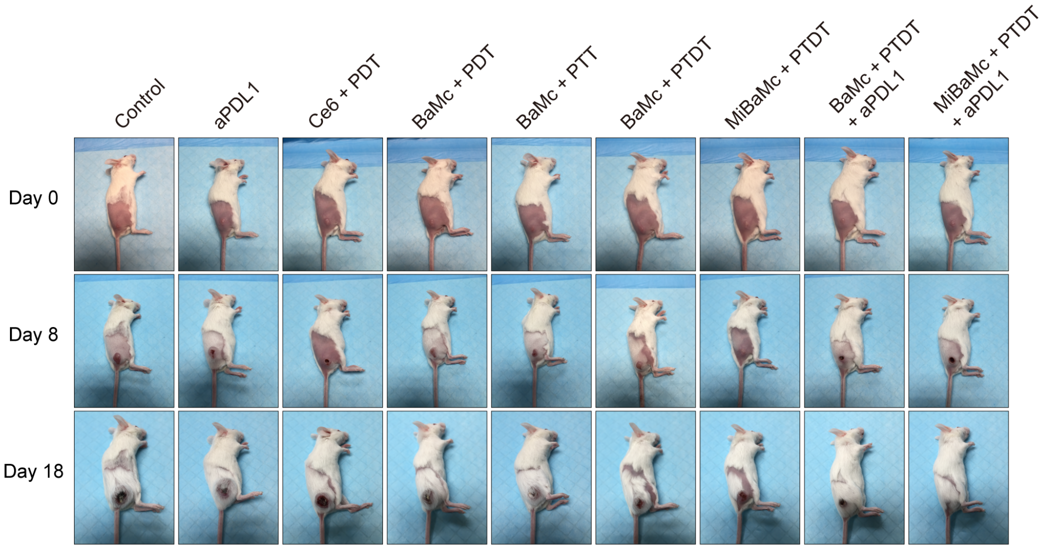

Supplementary Fig. 18. Photos of mice for 4T1 tumor-bearing mice. Representative photos of 4T1 tumor-bearing mouse from each group at different time points during the treatment. Supplementary Fig. 18 is relevant with Fig. 4d.

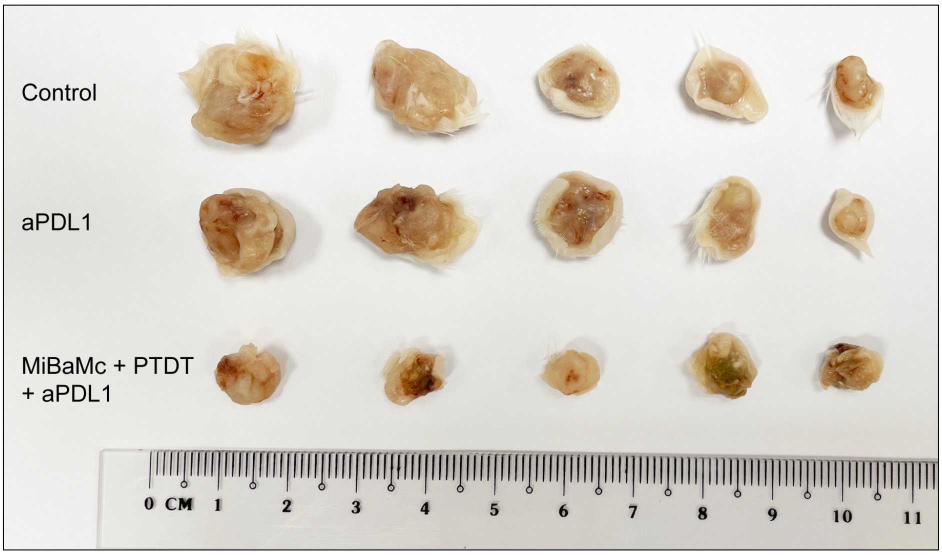

Supplementary Fig. 19. Photos of xenograft tumors from MC38 tumor-bearing mice. Photos of xenograft tumors after different treatments. Supplementary Fig. 19 is relevant with Fig. 4g.

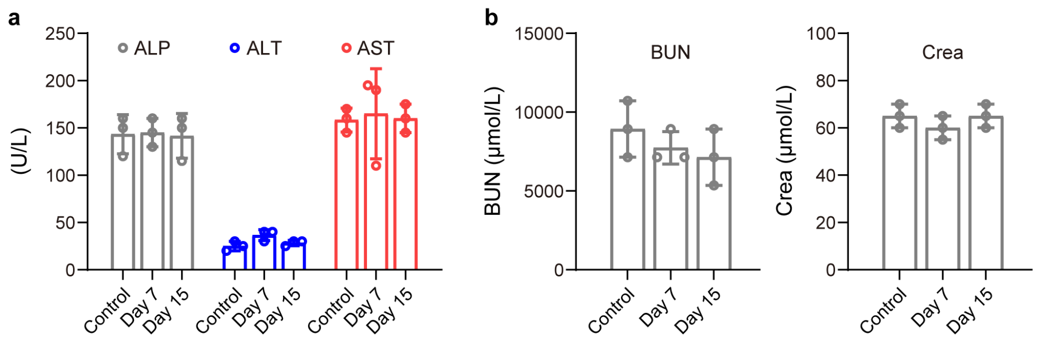

Supplementary Fig. 20. Liver and kidney function analysis of Balb/c mice at 7 and 15 days post-administration of MiBaMc. a, Blood biochemistry assays of liver function markers for alkaline phosphatase (ALP), alanine transaminase (ALT), and aspartate aminotransferase (AST). **b,** Blood biochemistry assays of kidney function markers for blood urea nitrogen (BUN) and creatinine (Crea). Data are presented as mean values ± SD (n = 3 mice).

Numbering: Control	Time: 20221221		Model: Whole blood
Project	Result	Unit	Reference range
WBC	5.0	10^9/L	0.8-6.8
Lymph#	2.9	10^9/L	0.7-5.7
Mon#	0.2	10^9/L	0.0-0.3
Gran#	1.7	10^9/L	0.1-1.8
Lymph%	57.9	%	55.8-90.6
Mon%	4.8	%	1.8-6.0
Gran%	37.3	%	8.6-38.9
RBC	6.72	10^12/L	6.36-9.42
HGB	106	g/L	110-143
HCT	34.2	%	34.6-44.6
MCV	50.9	fL	48.2-58.3
MCH	15.7	pg	15.8-19
MCHC	309	g/L	302-353
RDW	12.2	%	13-17
PLT	458	10^9/L	450-1590
MPV	7.3	fL	3.8-6.0
PDW	17.1		
PCT	0.370	%	

Supplementary Fig. 21. Blood routine analysis. Whole blood test of mice in control group injected with PBS (n = 3 mice).

Numbering: Day 1		Time: 20221221	Model: Whole blood
Project	Result	Unit	Reference range
WBC	8.8	10^9/L	0.8-6.8
Lymph#	6.2	10^9/L	0.7-5.7
Mon#	0.4	10^9/L	0.0-0.3
Gran#	2.2	10^9/L	0.1-1.8
Lymph%	70.4	%	55.8-90.6
Mon%	4.2	%	1.8-6.0
Gran%	25.4	%	8.6-38.9
RBC	9.52	10^12/L	6.36-9.42
HGB	144	g/L	110-143
HCT	46.4	%	34.6-44.6
MCV	48.8	fL	48.2-58.3
MCH	15.1	pg	15.8-19
MCHC	310	g/L	302-353
RDW	14.0	%	13-17
PLT	808	10^9/L	450-1590
MPV	6.7	fL	3.8-6.0
PDW	16.5		
PCT	0.541	%	

Supplementary Fig. 22. Blood routine analysis. Whole blood test of mice on day 1 post-injection with MiBaMc (n = 3 mice).

Numbering: Day 3	Time: 20221221		Model: Whole blood
Project	Result	Unit	Reference range
WBC	4.6	10^9/L	0.8-6.8
Lymph#	2.7	10^9/L	0.7-5.7
Mon#	0.2	10^9/L	0.0-0.3
Gran#	1.7	10^9/L	0.1-1.8
Lymph%	57.8	%	55.8-90.6
Mon%	5.4	%	1.8-6.0
Gran%	36.8	%	8.6-38.9
RBC	8.64	10^12/L	6.36-9.42
HGB	132	g/L	110-143
HCT	43.3	%	34.6-44.6
MCV	50.2	fL	48.2-58.3
MCH	15.4	pg	15.8-19
MCHC	303	g/L	302-353
RDW	14.5	%	13-17
PLT	678	10^9/L	450-1590
MPV	7.3	fL	3.8-6.0
PDW	17.2		
PCT	0.202	%	

Supplementary Fig. 23. Blood routine analysis. Whole blood test of mice on day 3 post-injection with MiBaMc (n = 3 mice).

Numbering: Day 7		Time: 20221221	Model: Whole blood
Project	Result	Unit	Reference range
WBC	5.1	10^9/L	0.8-6.8
Lymph#	3.8	10^9/L	0.7-5.7
Mon#	0.2	10^9/L	0.0-0.3
Gran#	1.5	10^9/L	0.1-1.8
Lymph%	55.3	%	55.8-90.6
Mon%	4.7	%	1.8-6.0
Gran%	35.4	%	8.6-38.9
RBC	8.53	10^12/L	6.36-9.42
HGB	126	g/L	110-143
HCT	41.0	%	34.6-44.6
MCV	51.4	fL	48.2-58.3
MCH	15.9	pg	15.8-19
MCHC	309	g/L	302-353
RDW	13.2	%	13-17
PLT	489	10^9/L	450-1590
MPV	7.0	fL	3.8-6.0
PDW	17.1		
PCT	0.342	%	

Supplementary Fig. 24. Blood routine analysis. Whole blood test of mice on day 7 post-injection with MiBaMc (n = 3 mice).

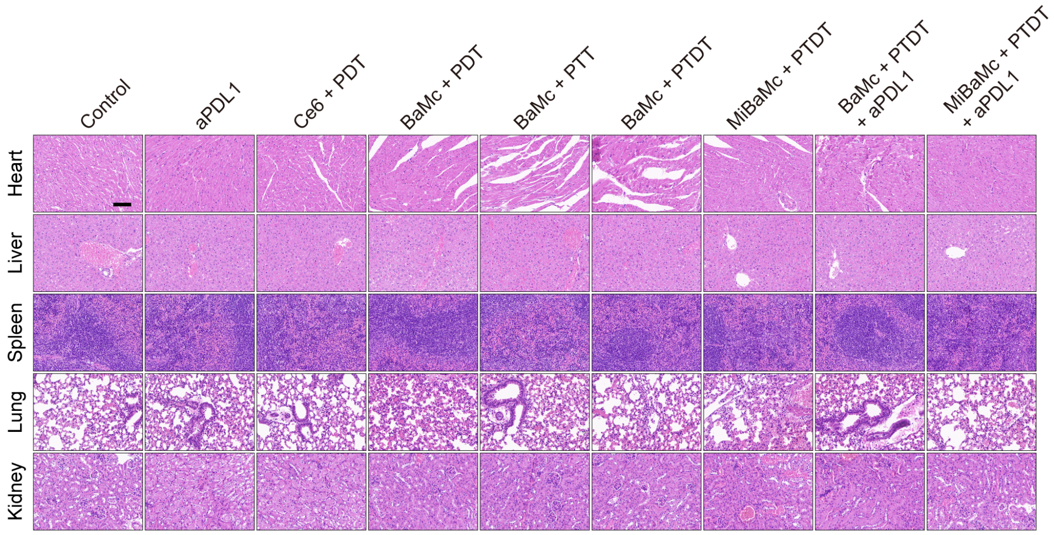

Supplementary Fig. 25. **Photographs of H&E stained sections of main organs from 4T1 tumor-bearing mice after different treatments.** Mice were treated with various formulations, and the main organs (heart, liver, spleen, lung, and kidney) were isolated after treatment for H&E staining (n = 3 mice). Scale bar, 100 µm.

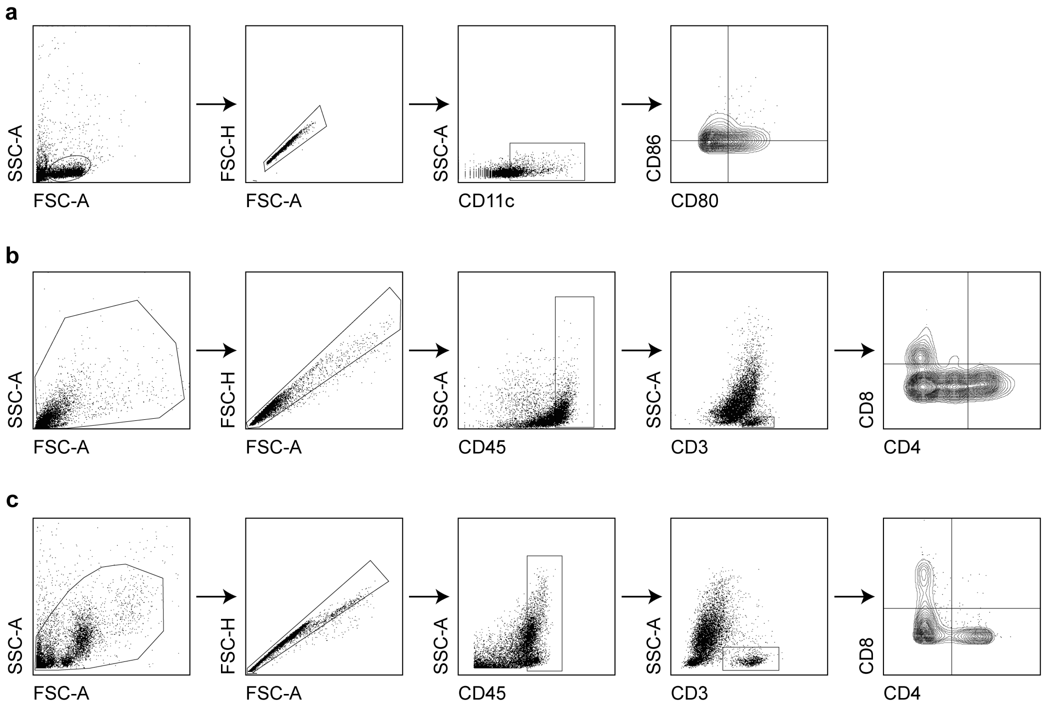

Supplementary Fig. 26. Gating strategies used for flow cytometry analysis of immune cells in vivo. a, Gating strategies to analyze matured DCs ($CD80^+$ and $CD86^+$) from tumor-draining lymph nodes of 4T1 tumor-bearing mice after each treatment. **b,** Gating strategies to analyze $CD3^+CD8^+$ T cells and $CD3^+CD4^+$ T cells as a percentage of $CD3^+$ lymphocytes from tumor tissues of 4T1 tumor-bearing mice after each treatment. **c,** Gating strategies to analyze splenic T lymphocytes ($CD3^+CD8^+$ and $CD3^+CD4^+$) as a percentage of $CD3^+$ lymphocytes from spleens in 4T1 tumor-bearing mice of 4T1 tumor-bearing mice after each treatment.

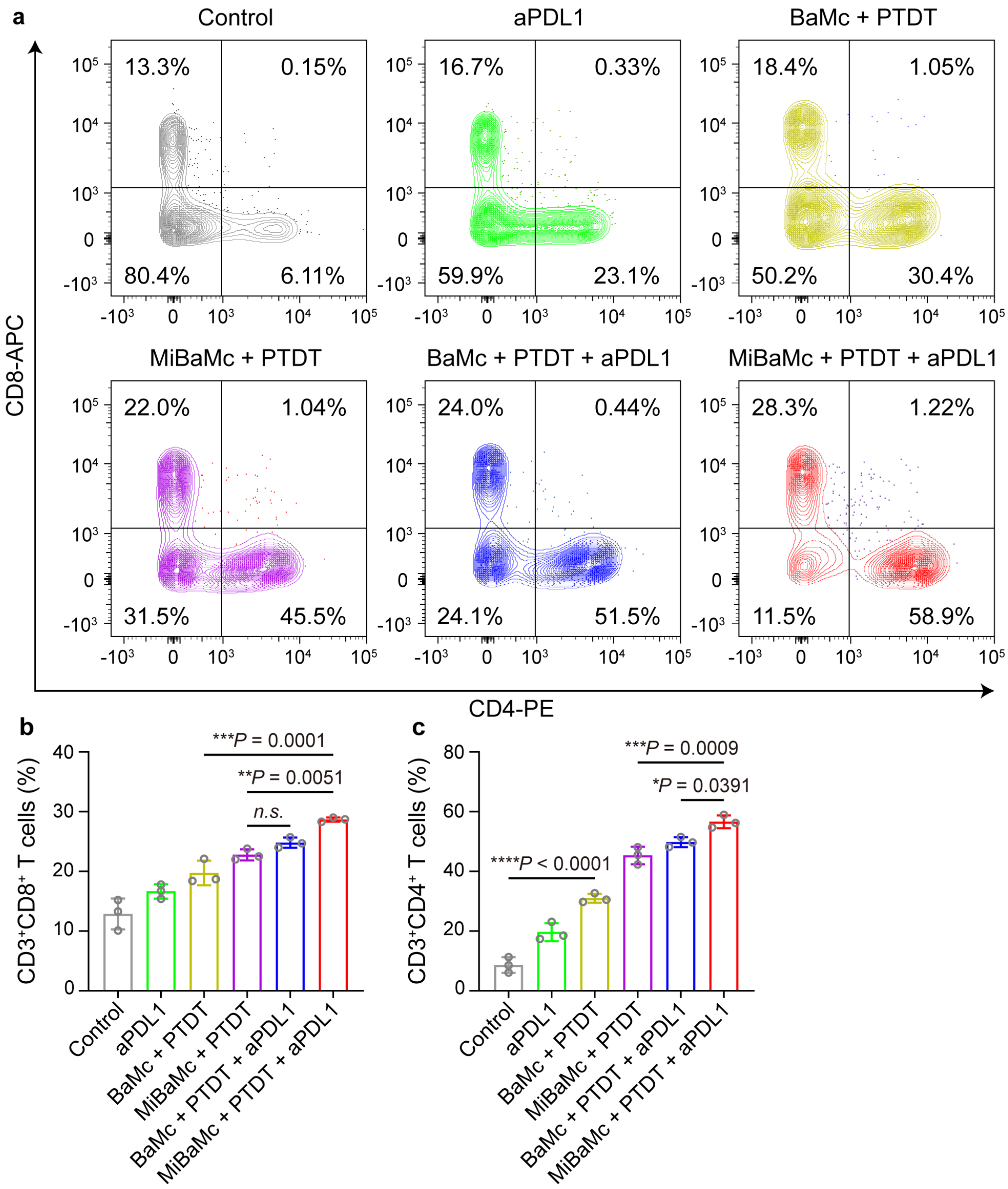

Supplementary Fig. 27. In vivo immune response in the spleen after different treatments. a, Representative flow cytometric plots of the $CD8^+$ T cells and $CD4^+$ T cells in 4T1 tumor-bearing spleen tissues after treatment of each formulation. Quantitative analysis of **b** $CD3^+CD8^+$ T cells and **c** $CD3^+CD4^+$ T cells as a percentage of $CD3^+$ T Lymphocytes based on flow cytometric results (**a**). Data are presented as mean values ± SD (n = 3 mice). Statistical analysis was conducted using one-way ANOVA with Tukey's tests. n.s. represents none of significance, $^*P < 0.05$, $^{**}P < 0.01$, $^{***}P < 0.001$, $^{****}P < 0.0001$.

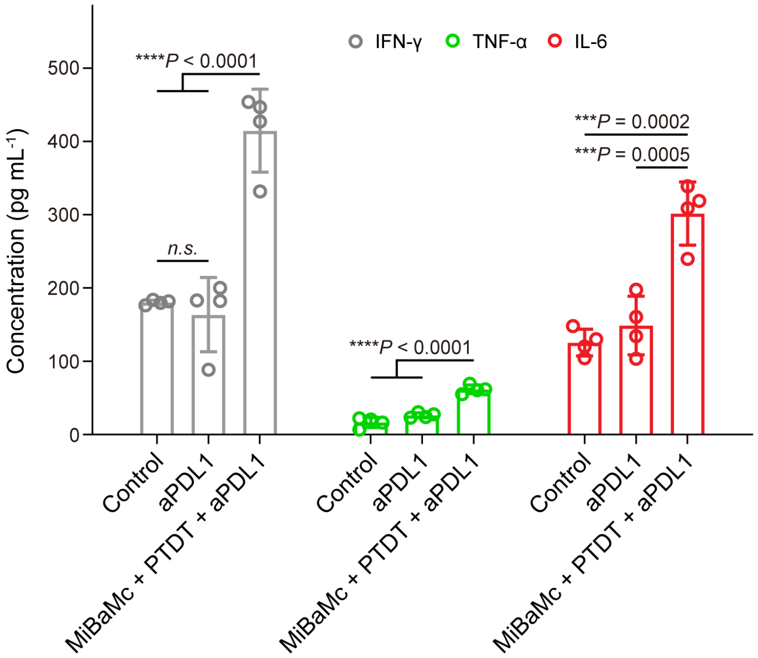

Supplementary Fig. 28. IFN-γ, TNF-α, and IL-6 expression profiles in serum of MC38 tumor-bearing mice after each treatment. Quantitative analysis of cytokine expression levels of IFN-γ, TNF-α, and IL-6 expression profiles in the serum of MC38 tumor-bearing mice after each treatment. Data are presented as mean values ± SD (n = 4 mice). Statistical analysis was conducted using one-way ANOVA with Tukey's tests. n.s. represents none of significance, $^{***}P < 0.001$, $^{****}P < 0.0001$.

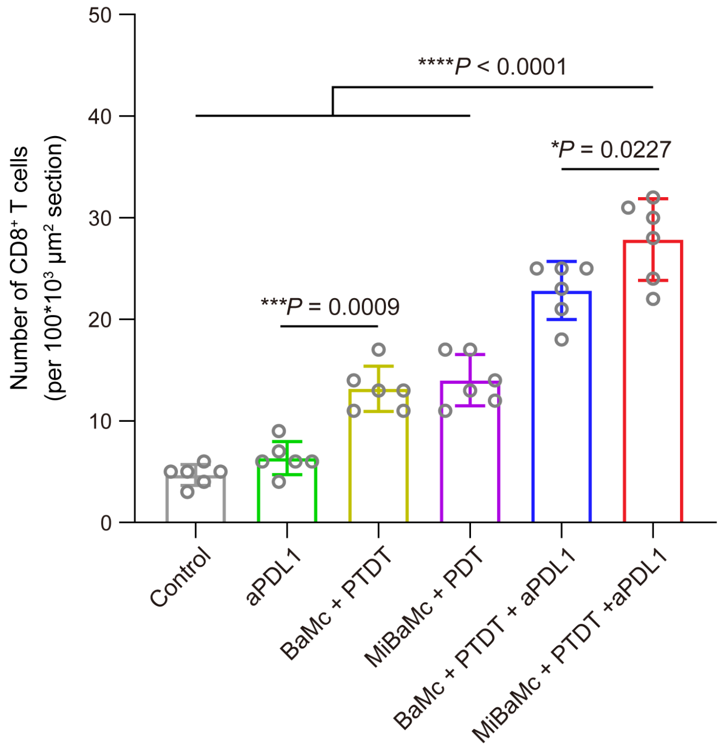

Supplementary Fig. 29. Number of $CD8^+$ T cells in tumor sections of 4T1 tumor-bearing mice after different treatments. Quantitative analysis of $CD8^+$ T cells in the sections of 4T1 tumor-bearing mice after each treatment. Data are presented as mean values ± SD (n = 6 samples). Statistical analysis was conducted using one-way ANOVA with Tukey's tests. $^{*}P < 0.05$, $^{***}P < 0.001$, $^{****}P < 0.0001$.

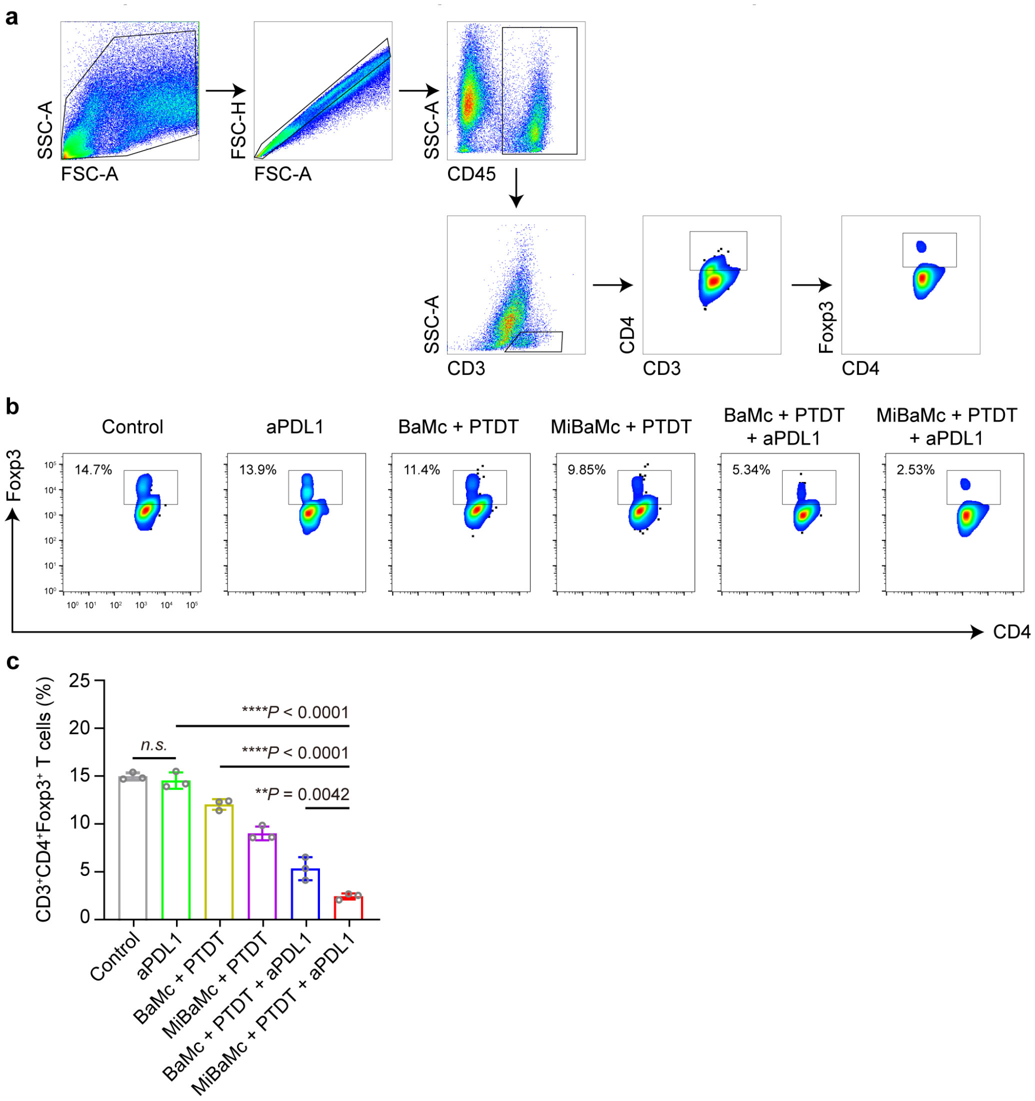

Supplementary Fig. 30. In vivo immune response in tumor tissues after different treatments. **a,** Gating strategy to analyze $CD3^+CD4^+Foxp3^+$ Tregs. **b,** Population of $CD3^+CD4^+Foxp3^+$ Tregs in the tumor tissues of each group a flow cytometric analysis according to flow cytometry. **c,** Quantitative analysis of the $CD3^+CD4^+Foxp3^+$ Tregs in each group. Data are presented as mean values ± SD (*n* = 3 mice). Statistical analysis was conducted using one-way ANOVA with Tukey's tests. n.s. represents none of significance, $^{**}P < 0.01$, $^{****}P < 0.0001$.

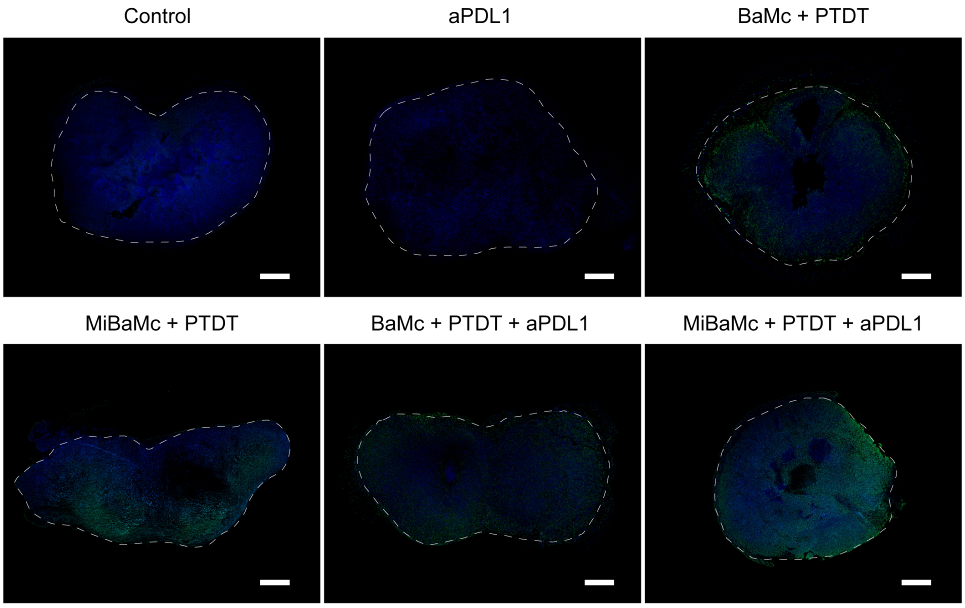

Supplementary Fig. 31. ROS fluorescence images of tumor slices. Representative ROS fluorescence images of tumor sections 12 h after different treatments. Green fluorescence signal represents ROS level. For ROS staining of tumor tissues: the mice were first intravenous administration of PBS and MiBaMc, 12 h later the mice were intraperitoneal administration of DCFH-DA (5 mg/kg), followed by irradiation or not. The tumors were surgically excised, and frozen tumor slices were prepared. Hoechst 33342 dye was applied to stain cell nuclei. Scale bar, 1000 μm. The fluorescent tumor slides were recorded using a confocal microscope (Nikon, Digital Eclipse C1 microscope system with NIKON DS-U3 controller). The experiments were repeated three times with similar results.

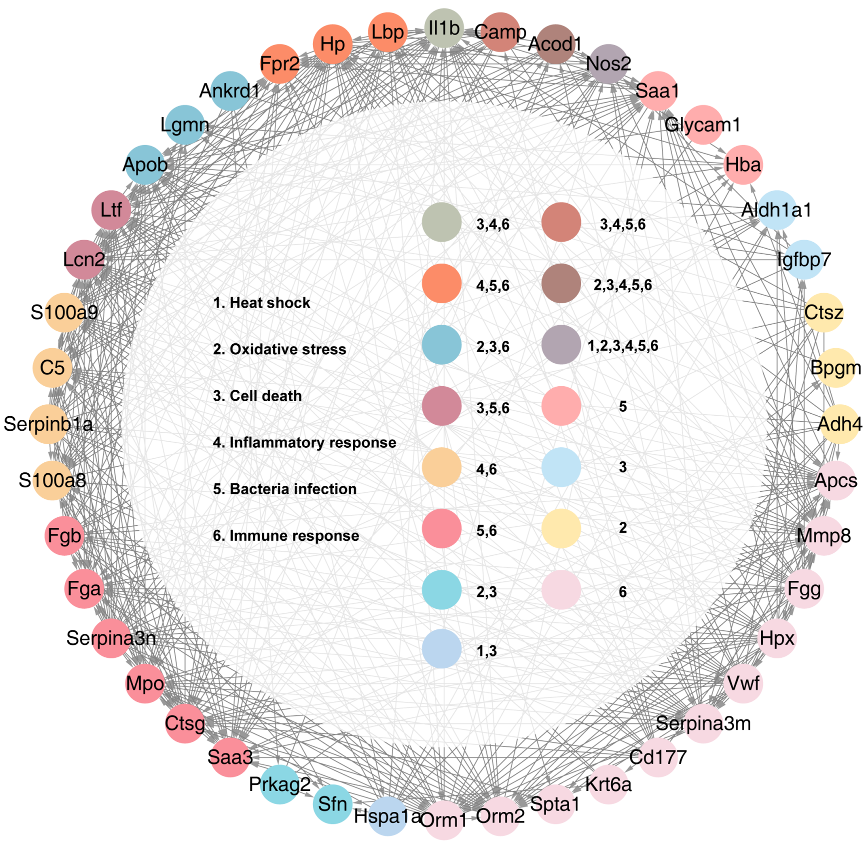

Supplementary Fig. 32. Proteomics analysis. Differentially expressed protein interaction networks. The circles with different colors represent the genes related to heat shock, oxidative stress, cell death, inflammatory response, bacterial infection, and immune response (refer to Fig. 6e). The line represents the protein-protein interaction.
