# Supplementary material for: Microbial synthesis of Prussian blue for potentiating checkpoint blockade immunotherapy
Source: Nat Commun. 2023 May 23;14:2943. doi: 10.1038/s41467-023-38796-9 (PMC10205718; doi:10.1038/s41467-023-38796-9)
Supplement: Supplementary file 3 — Reporting Summary [file 41467_2023_38796_MOESM3_ESM.pdf]

nature portfolio

Corresponding author(s): Yanli Zhao
Last updated by author(s): May 13, 2023

Reporting Summary

Nature Portfolio wishes to improve the reproducibility of the work that we publish. This form provides structure for consistency and transparency in reporting. For further information on Nature Portfolio policies, see our Editorial Policies and the Editorial Policy Checklist.

Statistics

For all statistical analyses, confirm that the following items are present in the figure legend, table legend, main text, or Methods section.

n/a	Confirmed	
☐	☒	The exact sample size (*n*) for each experimental group/condition, given as a discrete number and unit of measurement
☐	☒	A statement on whether measurements were taken from distinct samples or whether the same sample was measured repeatedly
☐	☒	The statistical test(s) used AND whether they are one- or two-sided<br>*Only common tests should be described solely by name; describe more complex techniques in the Methods section.*
☐	☒	A description of all covariates tested
☐	☒	A description of any assumptions or corrections, such as tests of normality and adjustment for multiple comparisons
☐	☒	A full description of the statistical parameters including central tendency (e.g. means) or other basic estimates (e.g. regression coefficient) AND variation (e.g. standard deviation) or associated estimates of uncertainty (e.g. confidence intervals)
☐	☒	For null hypothesis testing, the test statistic (e.g. *F*, *t*, *r*) with confidence intervals, effect sizes, degrees of freedom and *P* value noted<br>*Give P values as exact values whenever suitable.*
☒	☐	For Bayesian analysis, information on the choice of priors and Markov chain Monte Carlo settings
☒	☐	For hierarchical and complex designs, identification of the appropriate level for tests and full reporting of outcomes
☐	☒	Estimates of effect sizes (e.g. Cohen's *d*, Pearson's *r*), indicating how they were calculated

Our web collection on statistics for biologists contains articles on many of the points above.

Software and code

Policy information about availability of computer code

Data collection	Data of in vitro fluorescence imaging were collected with Carl Zeiss LSM800 confocal laser scanning microscopy. Data of in vivo and ex vivo fluorescence imaging were collected with the Living Image 4.3 software of IVIS fluorescence imaging system.
Data analysis	In vitro fluorescence imaging was analyzed using ZEN 2 (blue edition) software. In vivo and ex vivo fluorescence imaging was analyzed using the Living Image 4.3 software. Statistical analysis was calculated using GraphPad Prime 6.0. Flow cytometry results were analyzed by FlowJo version 10.0.7.

For manuscripts utilizing custom algorithms or software that are central to the research but not yet described in published literature, software must be made available to editors and reviewers. We strongly encourage code deposition in a community repository (e.g. GitHub). See the Nature Portfolio guidelines for submitting code & software for further information.

Data

Policy information about availability of data

All manuscripts must include a data availability statement. This statement should provide the following information, where applicable:

- Accession codes, unique identifiers, or web links for publicly available datasets
- A description of any restrictions on data availability
- For clinical datasets or third party data, please ensure that the statement adheres to our policy

The proteomics data are available at iProX under Project IPX0005125000. The authors declare that the data supporting the findings of this study are available within the article and its Supplementary Information. Source data are provided with this paper.

Human research participants

Policy information about studies involving human research participants and Sex and Gender in Research.

Reporting on sex and gender	*N/A*
Population characteristics	*N/A*
Recruitment	*N/A*
Ethics oversight	*N/A*

Note that full information on the approval of the study protocol must also be provided in the manuscript.

Field-specific reporting

Please select the one below that is the best fit for your research. If you are not sure, read the appropriate sections before making your selection.

☒ Life sciences ☐ Behavioural & social sciences ☐ Ecological, evolutionary & environmental sciences

For a reference copy of the document with all sections, see nature.com/documents/nr-reporting-summary-flat.pdf

Life sciences study design

All studies must disclose on these points even when the disclosure is negative.

Sample size	Animal experiments were performed under the permission of Guidelines for Care and Use of Laboratory Animals of the Institutional Animal Care and Use Committee of Nanyang Technological University (NTU-IACUC) with protocol number A19016. We used G*power analysis to calculate and ensure the sample sizes fulfill adequate power (p > 0.8). According to the experimental data and sample size (n), P value and effect size were calculated and the power was then calculated. If it is higher than 80%, demonstrating the sample size is adequate.
Data exclusions	No data was excluded from this study.
Replication	Experiments were repeated at least three independent experiments with similar results. All experiments were reproduced to reliably support conclusions stated in the manuscript.
Randomization	Mice in cages were selected randomly and then divided randomly into experimental groups before starting treatment. Other samples were also randomly grouped.
Blinding	Investigators were blinded to group allocation during the data collection and analysis.

Reporting for specific materials, systems and methods

We require information from authors about some types of materials, experimental systems and methods used in many studies. Here, indicate whether each material, system or method listed is relevant to your study. If you are not sure if a list item applies to your research, read the appropriate section before selecting a response.

Materials & experimental systems

n/a	Involved in the study	
☐	☒	Antibodies
☐	☒	Eukaryotic cell lines
☒	☐	Palaeontology and archaeology
☐	☒	Animals and other organisms
☒	☐	Clinical data
☒	☐	Dual use research of concern

Methods

n/a	Involved in the study	
☒	☐	ChIP-seq
☐	☒	Flow cytometry
☒	☐	MRI-based neuroimaging

Antibodies

Antibodies used

Rabbit anti-HMGB1 (cat no. ab79823, dilution of 1:250), rabbit anti-β-actin (cat no. ab213262, dilution of 1:1000), AF647-conjugated goat anti-rabbit IgG H&L (cat no. ab150079, dilution of 1:400) and AF488-conjugated goat anti-mouse IgG H&L (cat no. 150113, dilution of 1:400) were purchased from Abcam Inc. (Cambridge, CA, USA). Mouse anti-CRT (cat no. MA1-91034, dilution of 1:200), goat anti-rabbit IgG H&L HRP (cat no. 31466, dilution of 1:10000-1:20000), and goat anti-mouse IgG H&L HRP (cat no. 31431, dilution of 1:10000-1:20000) were purchased from ThermoFisher. ELISA kit for HMGB1 (cat no. EM0382) was purchased from Wuhan Fine Biotech Co., Ltd. ELISA kits for IFN-γ (cat no. 430804), TNF-α (cat no. 430901), and IL-6 (cat no. 431301), FITC-conjugated anti-CD80 (cat no. 104705, dilution of 1:50), PE-conjugated anti-CD86 (cat no. 105007, dilution of 1:200), APC-conjugated anti-CD11c (cat no. 117309, dilution of 1:80), AF700-conjugated anti-CD45 (cat no. 103127, dilution of 1:200), and anti-CD16/32 (cat no. 156603, dilution of 1:200), PE-conjugated anti-CD4 (cat no. 130310, dilution of 1:80), APC-conjugated anti-CD8a cat no. 100711, dilution of 1:80), FITC-conjugated anti-CD3 (cat no. 100204, dilution of 1:50), and AF647-conjugated anti-Foxp3 (cat no. 126407, dilution of 1:100) were purchased from Biolegend.

Validation

All antibodies were used in the study according to the profile of manufacturers. Antibody validation was validated by the supplier and confirmed in this study.

Eukaryotic cell lines

Policy information about cell lines and Sex and Gender in Research

Cell line source(s)

4T1 murine mammary carcinoma cell line, MC38 murine colon adenocarcinoma cell line, and HEK-293 human embryonic kidney 293 cell line were purchased from ATCC.

Authentication

The suppliers routinely authenticate the cell lines by short tandem repeat profiling though the cell lines were not authenticated by our laboratory.

Mycoplasma contamination

Mycoplasma test was negative by the supplier using PCR-based assay.

Commonly misidentified lines
(See ICLAC register)

No commonly misidentified cell line was used.

Animals and other research organisms

Policy information about studies involving animals; ARRIVE guidelines recommended for reporting animal research, and Sex and Gender in Research

Laboratory animals

Balb/c mice (female, 6-8 weeks) were purchased from InVivos Pte. Ltd. (Singapore).

Wild animals

No wild animals was used in this study.

Reporting on sex

This study did not involve sex consideration. Female mice were used according to literature reports in the field.

Field-collected samples

This study did not involve samples collected from field.

Ethics oversight

All animal experiments were reviewed and approved with the Guidelines for Care and Use of Laboratory Animals of the Institutional Animal Care and Use Committee of Nanyang Technological University (NTU-IACUC).

Note that full information on the approval of the study protocol must also be provided in the manuscript.

Flow Cytometry

Plots

Confirm that:

- [ ] The axis labels state the marker and fluorochrome used (e.g. CD4-FITC).
- [ ] The axis scales are clearly visible. Include numbers along axes only for bottom left plot of group (a 'group' is an analysis of identical markers).
- [x] All plots are contour plots with outliers or pseudocolor plots.
- [x] A numerical value for number of cells or percentage (with statistics) is provided.

Methodology

Sample preparation	For tumor tissues, samples were first digested for 2 h at 37 °C using type I collagenase (1 mg/mL), type IV collagenase (1 mg/mL), and DNase I (100 μg/mL). Then, the homogeneous samples were filtered via 70 μm nylon cell strainer. The red blood cells in tumor single cell suspension were removed using RBC Lysis Buffer according to the manufacturer's protocol. The tumor cells were washed with cell staining buffer and then stained with antibodies according to the manufacturer's protocols before conducting flow cytometry analysis. For lymph nodes, samples were directly ground and washed three times with cell staining buffer and stained with antibodies. For spleens, samples were ground and then the red blood cells were removed with RBC Lysis Buffer and washed three time with cell staining buffer for subsequent staining of corresponding antibodies.
Instrument	Fortessa X20 (BD Biosciences)
Software	FlowJo software package (version 10.0.7)
Cell population abundance	No cell sorting was performed in this study.
Gating strategy	A gate was drawn around the cells in the Supplementary Infornation. Cells were first determined through FSC-A vs. SSC-A to remove debris. Single cells were further determined through FSC-A vs. FSC-H.

- [x] Tick this box to confirm that a figure exemplifying the gating strategy is provided in the Supplementary Information.
